# Zimbabwean diabetics' beliefs about health and illness: an interview study

**DOI:** 10.1186/1472-698X-10-7

**Published:** 2010-05-12

**Authors:** Katarina Hjelm, Esther Mufunda

**Affiliations:** 1 School of Health and Caring Sciences, Linnaeus University, Växjö, S-351 95 Växjö, Sweden; 2 Department of Health Sciences, Zimbabwe Open University, Harare, Zimbabwe

## Abstract

**Background:**

Diabetes mellitus (DM) is increasing globally, with the greatest increase in Africa and Asia. In Zimbabwe a threefold increase was shown in the 1990s. Health-related behaviour is important in maintaining health and is determined by individual beliefs about health and illness but has seen little study. The purpose of the study was to explore beliefs about health and illness that might affect self-care practice and health care seeking behaviour in persons diagnosed with DM, living in Zimbabwe.

**Methods:**

Exploratory study. Consecutive sample from a diabetes clinic at a central hospital. Semi-structured interviews were held with 21 persons aged 19-65 years. Data were analysed using qualitative content analysis.

**Results:**

Health was described as freedom from disease and well-being, and individual factors such as compliance with advice received and drugs were considered important to promote health. A mixture of causes of DM, predominantly individual factors such as heredity, overweight and wrong diet in combination with supernatural factors such as fate, punishment from God and witchcraft were mentioned. Most respondents did not recognize the symptoms of DM when falling ill but related the problems to other diseases, e.g. HIV, malaria etc. Limited knowledge about DM and the body was indicated. Poor economy was mentioned as harmful to health and a consequence of DM because the need to buy expensive drugs, food and attend check-ups. Self-care was used to a limited extent but if used, a combination of individual measures, household remedies or herbs and religious acts such as prayers and holy water were frequently used, and in some cases health care professionals were consulted.

**Conclusions:**

Limited knowledge about DM, based on beliefs about health and illness including biomedical and traditional explanations related to the influence of supernatural forces, e.g. fate, God etc., were found, which affected patients' self-care and care-seeking behaviour. Strained economy was stated to be a factor of the utmost importance affecting the management of DM and thus health. To develop cost-effective and optimal diabetes care in a country with limited resources, not only educational efforts based on individual beliefs are needed but also considering systemic and structural conditions in order to promote health and to prevent costly consequences of DM.

## Background

Diabetes mellitus (DM) affects millions of people worldwide and its related complications continue to be of great concern [1]. Of those diagnosed with DM, 90-95% have type 2 DM. The number of affected people is estimated to double by 2025, with the greatest increase occurring in developing countries [2]. The regions with the greatest potential increase of DM in the future are Africa and Asia, where diabetes is estimated to become two to three times more common than today [1]. In Zimbabwe DM has been reported as the fifth among the ten most common diseases [3]. From 1990-1997 the prevalence of diabetes increased from 150 to 550 per 100,000 people [4]. Thus, the overall prevalence increased threefold. According to the Zimbabwe National Health Profiles (1996-1998) the number of new cases recorded in the ages 15 years and above rose from 2734 cases in 1996 to 5114 cases in 1998, which is an increase of 87% of recorded cases [5]. The increase of DM is related to changes of societies because of urbanization and industrialization, leading to changes in lifestyle from a 'traditional' and active life to a 'modern' sedentary life with unhealthy dietary habits and obesity in combination with increased longevity [2]. DM is thus a result of the collision between the modern lifestyle and our ancient genes built for a life as hunter-gatherer [6]. The main consequences of DM are reduced life expectancy, increased mortality and morbidity associated with development of complications with enormous costs, and thus DM will constitute a heavy burden for individuals as well as society [7,8].

The outcome of DM depends mainly on the patient's self-management, which is guided by individual and culturally determined beliefs about health and illness [9,10].

### Literature review

The literature search has not revealed any studies exploring the individual's own beliefs about health and illness in persons diagnosed with DM living in African countries with the exception of a comparison of Ugandan men and women [11]. Limited knowledge about DM and the body was indicated, and the majority did not know the cause of DM. Many attributed it to the influence of supernatural forces, which meant that limited self-care measures were used and health professionals were not consulted about health problems. Men focused on socio-economic factors, particularly the affordability of drugs, sexual function and lifestyle, while women valued well-being, support in daily life and household activities and had a higher risk awareness of DM. The indication of limited knowledge was in accordance with findings shown in previous investigations focusing on knowledge of DM in people in Cameroon and South Africa [12,13], and little understanding of the nature of DM, with reporting of a spectrum of different causes of DM in an investigation of the impact of the disease in Nigerians with DM [14], while in Ghanaians diagnosed with DM a blend of commonsense, scientized, and religious knowledge modalities that merged with biomedical goals, specifically drug and diet management [15].

When investigating health beliefs and stress with a quantitative approach [16] it was found that a number of patients suffered from considerable psychosocial stress mainly related to leisure time and physical complications resulting from the disease and sometimes associated with poor diabetic control. They concluded that health care staff needed to consider the self-perception of compliance held by patients in order to consolidate progress.

Previous studies comparing beliefs about health and illness in persons of different origin with DM have shown that Europeans cite various and more medically oriented causes of disease, for example heredity, obesity [9,10,17,18], whereas non-Europeans, e.g. North Africans, cited either stress or fate [17]. Middle-Easterners showed a similar pattern of beliefs to North Africans, with a more fatalistic view of DM in terms of factors lying beyond one's own control, such as fate and supernatural influence through the will of God or Allah (external locus of control; [9,10]. Although health was described similarly to freedom from disease, in Swedes, Ex-Yugoslavians and Arabs, three different self-care behaviours were demonstrated [9,10,18]. Swedes were active and had a healthy and controlled lifestyle. Ex-Yugoslavians highlighted enjoyment of life and had a passive self-care attitude. Arabs focused on mental well-being, adaptation to DM, and actively searched for information and had a lower threshold for seeking care [9,10,18]. Foreign-born persons perceived DM as less serious and knew less about their body and DM compared to Swedes.

### The study

The aim of the present study was to explore beliefs about health and illness that might affect self-care practice and health-care-seeking behaviour in persons diagnosed with diabetes mellitus living in Zimbabwe.

## Methods

### Design

An exploratory study design was used. Data were collected through semi-structured interviews, in 2004 and 2006, in order to give respondents freedom to express their views to reach a deeper understanding of the topic studied [19].

### Participants

A consecutive sampling procedure was used. Set criteria for inclusion were: diagnosis of DM, duration of DM >1 year, age >18 years, and without known psychiatric disorder. All respondents were managed in an outpatient diabetes clinic, at a governmental central hospital in an urban area also managing cases referred from rural areas. The staff included a leader who was a physician specialized in internal medicine and a team of general physicians, general nurses and nurse aides. The respondents were recruited by a nurse (the principal investigator) when visiting the clinic.

The sample comprised 21 persons (11 females and 10 males) aged 19-65 yrs (Md 48 yrs, see Table 1) born and living in Zimbabwe. The majority were treated with oral agents and reported complications related to DM from the eyes. Most were married, had gone through secondary school, and about half of the group were gainfully employed.

**Table 1:** Characteristics of the study population.

Variable	
Age (yr)*	48 (19-65)
	
Duration of DM *	10 (3-21)
	
Treatment (n)	
Oral agents	13
Insulin	8
Combination with insulin	0
	
Number of years spent in school*	10 (6-17)
Educational level (n)	
Primary	5
Secondary	10
Upper secondary (College)	4
University >2 years	2
	
Current working conditions (n)	
Gainfully employed	9
Unemployed	8
Pensioners	3
Students	1
	
Family circumstances (n)	
Married	13
Single	3
Divorced	2
Widowed	3
	
Complications related to DM (n)	
Eyes	10
Feet/lower extremity	6
Kidney	1
Hypertension	2
No complications	2

* Median (range).

### Ethical considerations

The study was approved by hospital ethics committee. Written informed consent was obtained from respondents in accordance with the Helsinki Declaration.

### Data collection

The interview started with standardized questions (15 minutes) focusing on socio-demographic and medical variables. Then a thematic interview guide with open-ended questions, including descriptions of common health problems related to DM, was used (for more details see [18]. Themes investigated were: content of health; factors of importance for health; causes, explanations and perceived consequences of DM; health-restorative activities; and care-seeking behaviours. The interview guide was based on findings and experiences from previous investigations [9,18] and peer-reviewed by GPs and nurses working in diabetes care. A pilot test of the interview guide was carried out with six persons (not included in the study), and minor changes to the wording and meaning were made.

Interviews were mainly held in Shona (n = 18) but also in English in some cases (n = 3) and led by a bilingual female nurse (second author) not involved either in the clinic or in management of the respondents. Shona and English are official languages in Zimbabwe [20].

The interviews were held in secluded rooms outside the clinic. The interviews lasted for 1-1.5 hours, were audio-taped and transcribed verbatim in English.

### Data analysis

Collection and analysis of data proceeded simultaneously for respondents until theoretical saturation was achieved [19]. After the interviews, the tapes were listened to and notes were taken about general findings, ideas and emerging themes. The endeavour in the analyses was to be open to as much variation as possible, and themes, patterns and contradictions were searched for [21]. By reviewing each line of the texts, topics were identified and the material was condensed into content categories (See example in Table 2). As previously described [9,10,18], the lay theory model of illness causation [22] and the model for health-care-seeking behaviour [23] were introduced and used as the main analytical categories [19]. Illness can be experienced as caused by factors in the individual, natural, social relations or in the supernatural sphere, and explanations of disease guide strategies for self-care, treatment of diseases and health-care-seeking behaviour [22]. Health care can be sought from the popular, professional or folk sector (i.e. family, friends or relatives, professionals, or folk-healers; [23].

**Table 2:** Causes of DM.

Main analytical category^a^	Factor	Numbers (n)	
Factors related to the individual	Heredity:	5	15^b^
	'....both of my parents are diabetic. I inherited it from them'		
			
	Wrong food:		
	'...especially foods that contain too much sugar'	3	14^6^
			
	Treatment with drugs:		
	'... the hydrochlorothiazide that I was taking'	5	5^b^
	'..maybe anti-hypertensive drugs'		
			
	Diseases of the pancreas: '...problems with the pancreas'	2	13^b^
			
	Obesity^b^		17^b^
			
	Wrong dietary habit^b^		14^b^
			
	Disease of the pancreas^b^		13^b^
			
Factors related to the social sphere	Stress^b^		8^b^
	Disturbances in relations to others^b^		5^b^
			
Factors related to the supernatural sphere	Supernatural thoughts^b^		3^b^
	Punishment from God, or gods^b^		3^b^
	Witchcraft^b^		4^b^
	Fate^b^		8^b^

^a^Analytical categories according to the lay model of illness causation by Helman (2007).

^b^Explanations of causes of DM evolved in discussions of a list of potential causes of DM.

To increase the trustworthiness of the results, the transcripts were analysed independently by two researchers [19]: a diabetes specialist nurse and a general nurse (first and second author) and the comparison showed high agreement. Content of categories were also checked by the first author.

## Results

### Beliefs about health

Health was described in terms of individual factors. The content was mainly focused on well-being, expressed in terms of freedom from disease and bodily pain, and being strong and fit

...free of disease...looking after oneself well... Involves a feeling of well-being with no bodily pain. (6)

...to be strong and fit...someone is strong without any illness. (2)

Measures used in order to feel well and experience good health were also described as individually related factors, and the two subcategories of lifestyle factors and looking after oneself emerged. Lifestyle factors mainly included conforming to the correct diet, but some others mentioned exercise. Hygiene, personal and environmental, compliance with drugs and diabetic diet, and avoiding factors with negative influence on health such as injuries, were included in looking after oneself:

I make sure that I get good body-building foods, maintain good body hygiene, make sure that my environment is clean and follow doctor's instructions on drug taking. (1)

Respondents predominantly talked about individual factors in terms of compliance with diet, drugs and following doctor's instructions and having check-ups as being important for their health as far as DM was concerned. There were also some who talked about lifestyle factors such as regular exercise and maintenance of good personal hygiene.

All respondents, with the exception of one, considered instrumental tangible support from their family members. Assistance mentioned was mainly in terms of material support with provision of money to buy food and drugs, equipment for self-monitoring of blood glucose (SMBG), and help with preparation of food and supervision of meals. Acceptance of a sick person as a diabetic and psychological support to live positively were also mentioned:

...help me to live positively, help with different things...provide money to buy medications and right diet. (10)

...understand my condition so that they will be able to assist when I fall sick. (11)

Health professionals were considered important for health mainly because they give information support about the disease, food preparation and medications. Many also discussed a combination of material and social support concerning health maintenance, expressed as control of the disease and screening for potential health problems, and counselling, e.g. encouragement and reminders to maintain good health. Finally, a few talked about emotional support in understanding the needs of a person with DM:

...give information...teach me those things that I am supposed to and not supposed to do...give more advice on food, especially alternatives that I can eat...inform on how to give injections. (1)

...these people understand me and my needs as a diabetic patient. (6)

...give advice on how to look after myself, food preparation and taking of drugs...monitor my blood sugar levels regularly (3)

Concerning factors harmful to health, it was mainly social factors in terms of a poor economic situation resulting in lack of food, money, expensive or unavailable drugs, and over-working that were mentioned. Some stated social relations such as being single or divorced and others discussed other diseases. One person talked about a harmful lifestyle with smoking.

...everything is just too expensive these days... (20)

...many factors...the way I am living these days...lack of food, lack of money to buy drugs and to come for reviews (1)

Awareness of bodily signs was the predominant way to know whether health became worse, e.g. mainly general body weakness or tiredness or passing too much urine, but also headache, loss of appetite, and drinking too much water were stated. Some talked about check-ups at hospital as a possibility to monitor health status:

...some symptoms that are noticed, for example passing lots of urine, feeling tired and having headaches. (10)

...can only tell when I come for reviews at the hospital...(1)

When discussing the influence of economy on health, all respondents unanimously expressed negative effects as they were unable to buy food or drugs or to go for reviews at the hospital.

...the present situation has become too hard to buy medications and food...I minimize coming for reviews because the situation forces me to. (1)

Measures claimed to improve health when diagnosed with DM were mainly prayers or a healing power (supernatural factors) and household remedies such as various herbs (garlic, zumbani (green leaves from a plant), teas, aloe) or cough remedies, paracetamol etc. (natural factors). However, there was also a group of people who said they had never used nature cure medicine or were not allowed by their church to use them. More than half of the respondents had been in contact with the diabetes association, to share information and disease experience with others, and there they received support and assistance in problem solving from experts, but some did not know about its existence (social factors):

...prayers are useful and can have a healing power (6)

...natural herbs, garlic to lower blood pressure and salicylate ointment for painful joints (17)

...people with diabetes come (to the diabetes association) and share information and ideas concerning solutions to their problems...get assistance from experts and health practitioners (3).

To maintain health and prevent complications related to DM, all respondents brought up individual factors. The majority talked about compliance, mainly in terms of diet with reduction of sugar intake and avoidance of overeating, but also about medication and check-ups. Avoiding stress and working hard to earn a living were also stated.

...avoid foods that contain too much sugar,...live a life that conforms to one with diabetes, go for regular check-ups, test my blood sugar levels and take pills and injections on time. (10)

### Beliefs about illness

All respondents, except one, had not suspected DM when they fell ill with the disease. Most described how they felt they were going to die, others suspected witchcraft and a few suspected other diseases such as malaria, HIV and AIDS. Several symptoms, for example, drinking a lot of water, passing a lot of urine, feeling of dying, general body weakness and being tired had been experienced. Most went to the professional sector, to see a physician, either at clinics, doctors' surgeries or hospitals, while a few sought help from the popular sector, such as husbands, wives or friends, and two went to the folk sector to see n'angas (traditional healers).

...experiencing a feeling of tiredness, loss of weight, dryness of the mouth and passing lots of urine...I got confused because I failed to understand what was happening....feeling of dying...went to seek advice from a sister-in-law (a nurse)...tested my urine...then taken to a private doctor. (9)

...started by excess loss of weight, drinking large amounts of water...and I thought I had been bewitched...was taken to a n'anga but I got worse...Taken to the doctor...later referred for admission to hospital. (5)

DM was perceived by some, and expressed in answers to open-ended questions, as being caused by heredity (individual factors), other drugs such as anti-hypertensives (natural factors) or wrong food (individual factors), while others related it to problems of the pancreas (individual factors), and some didn't know (see Table 2). When a list of potential causes of DM was presented, most talked about individual factors such as heredity, wrong diet and obesity, inactivity and diseases of the pancreas. Supernatural factors such as fate or the influence of God, witches or evil spirits were added as well as social factors such as stress. Thus, a mixture of causes were mentioned, although predominantly focused on individual factors such as heredity, overweight and wrong dietary habits in combination with supernatural factors lying outside the individual's own control in terms of fate, punishment from God and witchcraft.

When discussing what happens in the body when one gets DM and the function of the pancreas, in general limited knowledge was demonstrated. Most knew about the function of insulin in reducing blood sugar.

The majority of respondents perceived DM to be lifelong and persisting until death and only one person indicated that it could be controlled.

Knowledge about the action of drugs was limited. Most of the respondents were treated with oral agents and the others with insulin. Those who discussed the main effects of treatment in general stated regulation of blood sugar levels and a few talked about correction of eye sight or thirst.

Major problems as a consequence of DM were reported by about half of the studied group. These consisted of varying physical matters such as 'reduced vision', 'coma', 'swollen joints', and 'difficult deliveries', financial problems in terms of 'too expensive drugs' and in one case 'taking too frequent meals' (individual and social factors).

All respondents, with the exception of three persons, expressed fears related to DM because of different complications, mainly coma but also loss of sight, heart failure, stroke and diabetic foot ulcers, and in one case inability to buy drugs because of high costs (individual and social factors).

Most respondents experienced problems in their contact with health care staff who checked their DM. These were related to delays by doctors, not coming on time or having lack of time to explain things, and delays at the pharmacy or dispensary.

...doctors start their clinics late...see a lot of people at a time and are always in a hurry, so one does not get enough time to ask questions...delays at pharmacy...something has to be done. (10)

### Self-care and care-seeking pattern

Respondents spoke of being informed about the importance of reviewing the progress of DM by regular check-ups of sugar levels and drug doses by a physician.

About half of the group had been informed about foot care but with a limited content:

...advised that diabetic wounds take a long time to heal...should keep blood sugar low, avoid injuries and keep feet dry. (6)

Most respondents reported that advice had been given concerning the importance of SMBG in terms of using bodily signs, e.g. passing urine, drinking a lot, feeling sleepy, bitterness in the mouth etc., to know about blood glucose to avoid sudden changes in sugar and minimizing complications such as coma. Only one had the equipment for monitoring their blood glucose at home. Some had not received any advice at all.

When discussing recommendations for diet, most cited the importance of reducing the intake of carbohydrates, mainly sugar, and increasing the intake of vegetables. A few talked about reduction of fat and regular meals. Four persons had received initial information from a dietician.

Food without much sugar, less carbohydrates and lots of fruit and green vegetables. (10)

...not to eat sugary foods, take fats in small amounts, have snacks in between. (14)

As regards advice about exercise, most had been informed of the importance of regular exercise: 'to exercise regularly...to lose weight by walking or jogging' (4) and 'drugs to be taken as directed'(16).

All respondents in general reported they mainly followed advice received, as it prolongs life. Reasons for not following advice could be:

...I try but it's rather difficult...don't have money to buy drugs and necessary monitoring equipment. (7)

When discussing different common health problems related to DM, such as hyperglycaemia, repeated episodes of hypoglycaemia, gastrointestinal infection, common cold and pharyngitis, urinary infection, problems with the feet (crawling, burning, decreased sensitivity), spasm in the calf, hypertension, and albuminuria, most respondents had used self-care measures related to the individual sphere (e.g. changes of food intake, rest, wait-and-see), nature (medications or herbs) or the supernatural sphere (mainly prayers, sometimes holy water). When necessary, help was sought from the professional sector, mainly from doctors and in some cases from nurses at a hospital based clinic. Most respondents indicated difficulties in identifying the causes of the problems and said they were unsure or gave a variety of causes but often included an association with DM.

...I think it was because of my diabetes...I used herbal leaves to rub over painful joints, smoked the herbs...also prayed for relief of my body pains...it worked. In the second instance I visited my doctor. (8)

When discussing the occurrence of wounds on the feet, a pattern was evident where most respondents had sought help from the professional sector at hospitals (social factors) and related the problem to injuries.

## Discussion

This study is unique as it explores beliefs about health and illness in a group of Africans originating from Zimbabwe. The main results showed limited knowledge about the body and DM, health was expressed from a pathogenic point of view as freedom from disease, and individual factors such as compliance with advice received and drugs were considered important to promote and maintain health as well as to prevent deterioration of DM. When people experienced health problems related to DM, they used self-care measures to a limited extent, but when they did it was frequently a combination of individual measures, household remedies or herbs and prayers or holy water, and in some cases health care professionals were consulted for help. Poor economy was mentioned as being harmful to health, and perceived as a consequence of DM due to the need to buy expensive drugs and not being able to follow the recommended treatment regimen.

## Methods

A consecutive sampling procedure was used, giving all men and women visiting the clinic the same opportunity to participate in the study. The studied group mainly comprised persons with a secondary level of education, of working age, coming from both urban and rural areas of the country. Zimbabwe is considered to have one of Africa's best educational systems and thus the population is relatively well-educated [20]. Primary school is formally mandatory and followed by further education in secondary school. According to reports by UNESCO, four out of ten children started secondary school in 2002. About two thirds of the population live in rural areas but there is extensive migration into the bigger cities. Thus, the studied group appeared to represent the general population of Zimbabwe. Gender is not problematized in this first explorative step but will be further elaborated in an extended study.

Interviews were held mainly in Shona (n = 18) but also in English (n = 3), which are two of the three official languages in Zimbabwe [20]. Some 70-80% of the population belong to the Shona-speaking group and this is spoken in everyday life by the majority, but many also speak English, particularly in the cities. Ndebele is the third language, spoken in the south and western part of the country. The site where the study was carried out is situated in the central part of Zimbabwe.

In order to minimize the influence of different languages, the interview guide was pilot-tested in both languages, the interviews were conducted by a bilingual nurse, and the participants were offered a choice as to the language they preferred to use during the interview.

Triangulation of data by using different methods to gain knowledge by open-ended and closed questions, with probing for detailed beliefs and knowledge, made an in-depth understanding possible [19] and possibly also revealed more or less unconscious beliefs and thus avoided collecting the frequently criticized superficial knowledge obtained by using only structured interviews or questionnaires [24].

Results from qualitative studies might be seen as limited as regards the possibility of generalizing data from them [19]. However, the aim of the present study was to explore beliefs in a group of people diagnosed with type 2 DM and the focus was on disclosure of different perspectives and not on finding results generalizable to the whole population. Carefully collected and analysed qualitative data are transferable to other populations, or contexts similar in characteristics [19].

## Results

In this study beliefs about health were expressed from a pathogenic point of view [25] similar to what has been found in previous studies concerning beliefs about health and illness in Ugandan men and women [11] and in migrants with DM living in Sweden [9,10,18]. Economic factors were emphasized as a major influence on health and the ability to comply with advice received about the management of DM. Underlying living conditions might thus be a barrier to adequate management of the disease, as well as an underlying cause to it [26], and need to be considered in diabetes care. A conflict between willingness to comply and ability to comply was demonstrated, and this is related to the current economic crisis in Zimbabwe. The crisis started in the late 1990s and has entailed a social deterioration with increasing poverty, poorer public health, lack of food etc [20]. In 2006 about 80% of the population of working age were unemployed, 70-80% of the Zimbabweans are estimated to live in poverty, and about 4.3 million people were in need of food aid according to the United Nations World Food Programme in 2005. Similar findings have been shown among Ugandans [11]. The diabetes epidemic, particularly its distribution, is argued [26] to be produced by poverty. The cumulative effects of structural constraints on healthy lifestyles and lack of a right to adequate medical care, are results of poverty leading to diabetes and its complications, and to disparities among social groups. However, the differences are avoidable, unjust and unnecessary. The roots of the pandemia lie in inequalities in social power and the solutions required are structural.

Beliefs about health and illness were mainly related to factors in the individual combined with factors in nature and the supernatural sphere as regards ways to improve health or measures to restore health after having problems or being ill. Health care was consulted to a limited extent and with few exceptions in the professional sector when needed.

Health-related behaviour thus did not correspond to what has previously been described in persons of non-western origin [22,23]. Non-westerners have been described as focusing on the social or supernatural spheres to explain illness causation, and contacting family or friends in the popular sector first when in need of care and then turning to traditional healers in the folk sector, in contrast to westerners who emphasize factors in the individual or nature and mainly consult the professional health care sector in the event of problems. As in previous investigations [9-11,18], the results do not correspond to the theoretical models [22,23] chosen for analysing data. The differences might be explained by, for example, dissimilarities in health care systems in different countries and restricted empirical testing of explanatory models [9,10], which further emphasizes the need to avoid crude generalizations about people's beliefs and instead to probe for and verify the individual's own perspective. However, the models do play an important role as a framework when searching for different perspectives.

Findings from open-ended questions showed that many respondents were unsure of the cause of DM, while some stated biomedical explanations (antihypertensive drugs, pancreatic disease). When adding results from discussions of a list of potential causes, respondents mainly mentioned individual factors such as obesity and unhealthy diet but also supernatural causes such as fate, punishment from God, spirits etc. lying outside the person's own control. Thus, the pattern appears to be more similar to what has been found in Europeans citing medically oriented causes, e.g. heredity and obesity [9,10,17,18] but also a fatalistic view discussing factors beyond a person's own control such as fate, the will of God etc. (an external locus of control; shown in Ugandans [11], North Africans [17] and Middle-Easterners [9,10]. Thus, a mixture of explanations was found that might be related to limited knowledge about DM, also evident in discussions concerning the pathophysiology of DM, action of drugs, and many were unable to identify the disease at the onset and suspected other diseases such as HIV, AIDS, malaria etc. The results confirmed previous findings in Ugandans [11] and concerning limited knowledge about DM in Africans [12-15]. The limited knowledge about DM is also reflected in self-care measures undertaken to restore and maintain health, as many had problems in identifying the causes of their health problems and frequently used supernatural measures (prayers, holy water) and natural factors (herbal remedies). Frequent use of folk medicine and visits to traditional healers have previously been shown in Nigerians [27], and the use of complementary alternative medicine might be explained by its accessibility in countries where this is part of and recognized in the existing health care system, as e.g. in Zimbabwe [5]. However, knowledge deficit might be related to the organization of diabetes care, as many expressed dissatisfaction in contact with health care related to delays by doctors and disruptions in the continuity of care, and limited time for consultations affecting the encounters with physicians and the ability to pose questions and receive information about the management of DM. Another factor influencing knowledge might be the fact that staff managing the clinic were not specialized in the area of diabetes care, which has previously been shown to affect beliefs about health and illness in persons with DM [28]. In a study in South Africa, lack of knowledge and need for further education related to diabetes care have been identified as barriers to optimal diabetes care [29]. The health care system in the present study did not serve the diabetic persons and introduced barriers to health. Structural conditions in the health care system and the society thus, influenced individual beliefs about health and illness and the results confirm the importance of considering that population health is not only related to life-style but is also tied into concrete conditions of existence and a broader socio-economic context [30]. Health promotion is an activity concerned with improving living conditions and empowering communities to gain control over the determinants of health. Reports from Havana [31] demonstrate that a different approach to care in terms of empowering patients with skills and perceptions to cope with diabetes in a Continuing Interactive Education in group discussions, cultural activities, dining out, and the like can be effective in an impoverished situation. The patients might need a complete new setting to learn to live with the disease under their conditions.

## Conclusions

Although limited knowledge was demonstrated there were indications of a potential to develop an attitude for improving knowledge and self-care if this is supported by relevant information about DM and improved socioeconomic conditions, as many respondents emphasized a willingness to comply with advice received, knew that the disease is life-long and had adequate fears of developing complications related to DM. Thus, it is important is to organize health care in a way that elicits individual beliefs about health and illness in persons with DM and then supports and provides the individual with appropriate information to strengthen the patient's self-care capability to become an active participant and partner in diabetes care [13]. In a country and life situation with a highly strained economic situation, as for example in Zimbabwe today [20] and in many other developing countries, health care needs to switch from predominantly focusing on disease control and compliance with medication to a holistic attitude starting from an individual perspective but also considering social determinants of health [26,30] to promote health and prevent DM and complications related to DM [26] in order to decrease the burden of the disease in light of the overarching pandemic of DM [2,8]. Health promotive work need to focus on the importance of structural changes with improving living conditions and development of quality care easily accessible and equally delivered [30]. The importance of patient education needs to be considered and the health care organization developed by giving room for the patient's thoughts and questions [9-11,18], considering the need for specialist training of staff working in diabetes care [27], choosing an appropriate category of staff to provide patient education, and finding optimal strategies for teaching, as regards both methods and point in time and also to empower them and gain control over the determinants of health [26,30].

In conclusion, this study found limited knowledge about DM based on beliefs about health and illness, including both modern biomedical and traditional explanations related to the influence of supernatural forces such as fate, God, spirits, witchcraft etc., that influence health-related behaviour and thus self-care measures and care-seeking behaviour. A factor of the utmost importance affecting management of DM was the strained economic situation, and in order to develop cost-effective and optimal diabetes care in a country with limited economic resources and 'the double burden of disease' [2,7,8] not only educational efforts based on individual beliefs are needed. Also the influence of structural factors in the health care system and society [26,30] need to be considered as regards both patients with DM and health care staff working in diabetes care, in order to promote health and prevent the occurrence of costly complications related to DM.

## Competing interests

The authors declare that they have no competing interests.

## Authors' contributions

KH and EM performed the study concept and design; EM did the data collection; EM and KH analysed and interpreted data; KH drafted the manuscript; EM and KH made final critical revisions for important intellectual content of the manuscript. All authors read and approved the final manuscript.

## Pre-publication history

The pre-publication history for this paper can be accessed here:

http://www.biomedcentral.com/1472-698X/10/7/prepub

## References

[B1] WHO World Health Organisation Chronic Diseases: A vital investment 2005 Geneva: World Health Organisation

[B2] Hjelm K Mufunda E Nambozi G Kemp J Preparing nurses to face the pandemic of diabetes mellitus: a literature review Journal of Advanced Nursing 2003 41 424 434 10.1046/j.1365-2648.2003.02548.x 12603567

[B3] Mudiayi TK Onyanga-Omara A Gelman ML Trends of morbidity in general medicine at United Bulawayo Hospitals, Bulawayo, Zimbabwe Cent Afr J Med 1997 43 213 219 9431757

[B4] Mufunda J Chatora R Ndanbakuwa Y Nyarango P Chitanba J Kasia A Sparks H Prevalence of noncommunicable diseases in Zimbabwe: Results from analysis of data from the National Central Registry and urban survey Ethnicity & Disease 2006 16 718 22 16937610

[B5] Ministry of Health and Child Welfare, Zimbabwe National Health Strategy for Zimbabwe 1997-2007 1999 Ministry of Health and Child welfare, Zimbabwe

[B6] Zimmet P Globalization, coca-colonization and the chronic disease epidemic: can the Doomsday scenario be averted? Journal of Internal Medicine 2000 247 301 310 10.1046/j.1365-2796.2000.00625.x 10762445

[B7] Zimmet P Alberti KG Shaw J Global and societal implications of the diabetes epidemic Nature 2001 414 782 787 10.1038/414782a 11742409

[B8] Zimmet PZ Alberti GMM World Health Organisation Report Introduction: Globalization and the non-communicable disease epidemic Obesity 2006 14 1 3 10.1038/oby.2006.1 16493116

[B9] Hjelm K Bard K Nyberg P Apelqvist J Religious and cultural distance in beliefs about health and illness in women with diabetes mellitus of different origin living in Sweden International Journal of Nursing Studies 2003 40 627 643 10.1016/S0020-7489(03)00020-8 12834928

[B10] Hjelm K Bard K Nyberg P Apelqvist J Beliefs about health and illness in men with diabetes mellitus of different origin living in Sweden Journal of Advanced Nursing 2005 50 47 59 10.1111/j.1365-2648.2004.03348.x 15788065

[B11] Hjelm K Nambozi G Beliefs about health and illness: a comparison between Ugandan men and women living with diabetes mellitus Int Nurs Rev 2008 55 434 441 10.1111/j.1466-7657.2008.00665.x 19146555

[B12] Kiawi E Edwards R Shu J Unwin N Kamadjeu R Mbanya JC Knowledge, attitudes and behaviour relating to diabetes and its main risk factors among urban residents in Cameroon: A qualitative survey Ethnicity & Disease 2006 16 503 509 17682255

[B13] Shilubane HN Potgieter E Patients' and family members' knowledge and views regarding diabetes mellitus and its treatment Curationis 2007 30 58 65 17703823 10.4102/curationis.v30i2.1074

[B14] Famuyiwa OO Edozien EM Ukoli CO Social, cultural and economic factors in the management of diabetes mellitus in Nigeria Afr J Med Sci 1985 14 3-4 145 54 3004173

[B15] De-Graft Aikins Living with diabetes in rural and urban Ghana: A critical social psychological examination of illness action and scope for intervention J Health Psychol 2003 8 557 572 10.1177/13591053030085007 19177717

[B16] Bopape M Peltzer K Health beliefs and stress among non-insulin dependent diabetes outpatients in a rural teaching hospital in South Africa Health SA Gesondheid 2002

[B17] Dechamp-Le-Roux C Valensi P Assad N Sislian P Attali JR Croyances des diabétiques sur l'étiologie de leur maladie. Influence de l'éthnie. (Aetiological beliefs in diabetic patients. Influence of ethnic origin) Diabete-Metab 1990 16 207 212 2210015

[B18] Hjelm K Nyberg P Apelqvist J Beliefs about health and illness essential for self-care practice: a comparison of migrant Yugoslavian and Swedish diabetic females Journal of Advanced Nursing 1999 30 1147 1159 10.1046/j.1365-2648.1999.01167.x 10564414

[B19] Patton MQ Qualitative research and evaluation methods 2004 4 London: Sage Publications

[B20] SIDA Landguiden Uganda, Afrika. (The land guide of Uganda, Africa) 2007 http://www.landguiden.se/pubCountryText.asp?country_id=178&subject_id=0 20081201

[B21] Mayring P Qualitative Content Analysis Forum: Qualitative Sozialforschung/Forum: Social Research 2000 1 2 http://www.qualitative-research.net/index.php/fqs/article/viewArticle/1089/2385 On-line journal

[B22] Helman C Culture, Health and Illness 2007 London: Butterworth & Co (Publishers), Ltd

[B23] Kleinman A Patients and healers in the context of culture 1980 London, University of California Press, Ltd

[B24] Stuart L Wiles PG A comparison of qualitative and quantitative research methods used to assess knowledge of foot care among people with diabetes Diabetic Medicine 1997 14 785 791 10.1002/(SICI)1096-9136(199709)14:9<785::AID-DIA466>3.0.CO;2-0 9300230

[B25] Antonovsky A Unraveling the mystery of health 1987 San Francisco: Jossey-Bass Inc

[B26] Chaufan C What does justice have to do with it? A bioethical and sociological perspective on the diabetes epidemic Advances in Medical Sociology 2008 9 269 300 full_text

[B27] Mammah Popoola M Living with diabetes. The holistic experiences of Nigerians and African Americans Holistic Nursing Practice 2005 19 10 16 15736725 10.1097/00004650-200501000-00006

[B28] Hjelm K Berntorp K Frid A Aberg A Apelqvist J Beliefs about health and illness in women managed for gestational diabetes in two organisations Midwifery 2008 4 168 82 10.1016/j.midw.2006.12.008 17360084

[B29] Haque M Emerson Hayden S Dennison C Navsa M Levitt N Barriers to initiating insulin therapy in patients with type 2 diabetes mellitus in public-sector primary health care centres in Cape Town SAMJ 2005 95 798 802 16341336

[B30] Raphael D Grasping at straws: a recent history of health promotion in Canada Critical Public Health 2008 4 483 495 10.1080/09581590802443604

[B31] García R Suárez R Diabetes education in the elderly: a 5-year follow-up of an interactive approach Patient Education and Counselling 1996 29 87 97 10.1016/0738-3991(96)00937-8 9006225

